# Knockdown of NADK promotes LUAD ferroptosis via NADPH/FSP1 axis

**DOI:** 10.1007/s00432-024-05752-z

**Published:** 2024-05-03

**Authors:** Xiangpeng Meng, Fang Peng, Shijie Yu, Xinming Chi, Wenchi Wang, Shujuan Shao

**Affiliations:** 1 Liaoning Key Laboratory of Proteomics, Dalian Medical University, Dalian, 116044 China; 2 Department of Pathologic, The Second Hospital of Dalian Medical University, Dalian, 116011 China

**Keywords:** Ferroptosis, NADK, NADPH, FSP1, LUAD

## Abstract

**Background:**

Lung cancer is a serious threat to human health and is the first leading cause of cancer death. Ferroptosis, a newly discovered form of programmed cell death associated with redox homeostasis, is of particular interest in the lung cancer, given the high oxygen environment of lung cancer. NADPH has reducing properties and therefore holds the potential to resist ferroptosis. Resistance to ferroptosis exists in lung cancer, but the role of NADK in regulating ferroptosis in lung cancer has not been reported yet.

**Methods:**

Immunohistochemistry (IHC) was used to analyse the expression of NADK in 86 cases of lung adenocarcinoma(LUAD) and adjacent tissues, and a IHC score was assigned to each sample. Chi-square and kaplan-meier curve was performed to analyse the differences in metastasis and five-year survival between the two groups with NADK high or low scores. Proliferation of NADK-knockdown LUAD cell lines was detected in vivo and vitro. Furthermore, leves of ROS, MDA and Fe^2+^ were measured to validate the effect and mechanism of NADK on ferroptosis in LUAD.

**Results:**

The expression of NADK was significantly evaluated in LUAD tissues as compared to adjacent non-cancerous tissues. The proliferation of NADK-knockdown cells was inhibited both in vivo and vitro, and increasing levels of intracellular ROS, Fe^2+^ and lipid peroxide products (MDA) were observed. Furthermore, NADK-knockdown promoted the ferroptosis of LUAD cells induced by Erastin/RSL3 by regulating the level of NADPH and the expression of FSP1. Knockdown of NADK enhanced the sensitivities of LUAD cells to Erastin/RSL3-induced ferroptosis by regulating NADPH level and FSP1 expression.

**Conclusions:**

NADK is over-expressed in LUAD patients. Knockdown of NADK inhibited the proliferation of LUAD cells both in vitro and in vivo and promotes the Erastin/RSL3-induced ferroptosis of LUAD cells by down-regulating the NADPH/FSP1 axis.

**Supplementary Information:**

The online version contains supplementary material available at 10.1007/s00432-024-05752-z.

## Introduction

Globally, lung cancer ranks second in cancer incidence and continues to lead in cancer mortality. Approximately estimated 1.8 millions people die from lung cancer each year, which is about 2 times the mortality of colorectal cancer, the second leading cause of cancer-related deaths (Sung et al. 2021). According to the histopathological classification, non-small cell lung cancer (NSCLC) is the most prevalent one, accounting for about 85% of lung cancer, of which lung squamous cell carcinoma, especially lung adenocarcinoma (LUAD) are the two major subtypes of NSCLC (Herbst et al. 2018; Molina et al. 2008). LUAD shows a higher incidence than lung squamous cell carcinoma (Roy-Chowdhuri 2021; Ruiz-Cordero and Devine 2020).In non-smoking populations, air pollutants such as PM2.5 are more likely to cause LUAD (Hill et al. 2023). Therefore, study of the pathogenesis and clinical treatment methods of LUAD is a critical issue that needs to be solved urgently.

Ferroptosis is a newly identified form of programmed cell death, triggered by an imbalance in cellular redox homeostasis, which is distinct from apoptosis (Cao and Dixon 2016; Dixon et al. 2012; Stockwell 2022; Yang and Stockwell 2016). Ferroptosis has reported to be closely related to various diseases, and resistance to ferroptosis is of great significance for the development and progression of tumor (Chen et al. 2021; Jiang et al. 2021; Yan et al. 2021). Studies have shown that tumor cells can evade immune-mediated killing by resisting to ferroptosis (Chen et al. 2023; Friedmann Angeli et al. 2019). Furthermore, ferroptosis may also be related to the resistance to traditional anticancer drugs. For instance, Metallothionein-1G (MT-1G) facilitates sorafenib resistance in hepatocellular carcinoma (HCC) cells, while knockdown of MT-1G can deplete glutathione and induce ferroptosis in sorafenib-treated HCC cells, thus restoring sensitivity of HCC cells to sorafenib (Sun et al. 2016). These findings suggest that targeting ferroptosis could synergize with other anticancer drugs and offer a new direction for overcoming tumor drug resistance.

In lung cancer, several proteins have been identified to modulate the ferroptosis process and influence tumor fates, among which GPX4 and FSP1 can reduce the sensitivity of lung cancer to ferroptosis by enhancing cellular antioxidant capacity. GPX4 combats ferroptosis by reducing lipid peroxides with reduced glutathione (Alim et al. 2019; Kim et al. 2023a), while FSP1 utilizes NAD(P)H and oxidative CoQ_10_ to synthesize NAD(P)^+^ and reduced CoQ_10_, thereby inhibiting ferroptosis (Bersuker et al. 2019; Doll et al. 2019). However, it was unclear whether NADH or NADPH served as the reducing equivalent in this process. NADH and NADPH share similar structure, with the primary distinction being the esterification of the 2’-hydroxyl group in the adenosine moiety of NADPH with phosphate. Recently, it has been reported that FSP1 exclusively utilizes NADPH, not NADH, for the reduction of CoQ10 (Zhang et al. 2023). NADPH has reducing properties and therefore holds the potential to resist ferroptosis (Ying 2008).

NADK, a key enzyme that catalyzes the conversion of NAD^+^ to NADP^+^, is one of the most important sources of intracellular NADPH (Hoxhaj et al. 2019; Pollak et al. 2007; Rather et al. 2021). It has been reported that *NADK* can regulate tumor cell proliferation and metastasis by modulating cellular metabolism (Ilter et al. 2023; Schild et al. 2021; Tedeschi et al. 2016). Therefore, we propose that NADK may be involved in tumor cell resistance to ferroptosis. However, no relevant studies have been published to date.

In this study, we aimed to explore whether NADK could serve as a potential target for sensitizing LUAD to ferroptosis. Our study showed that the expression of NADK was elevated in LUAD tissue samples compared to adjacent non-cancerous tissues and high NADK expression correlated with poor prognosis. Knockdown of NADK inhibited LUAD cell proliferation in vivo and vitro, and enhanced sensitivity of LUAD cells to ferroptosis inducer. Mechanistically, knockdown of NADK led to decreased levels of NADP(H) and FSP1. These findings provide evidence for NADK as a potential target for clinical treatment of LUAD.

## Materials and methods

### Cell culture and reagents

Human LUAD cell lines A549 and H1299 as well as human embryonic kidney cells HEK293T were obtained from the American Type Culture Collection (ATCC). LUAD cells were cultured in RPMI 1640 (Gibco, Amercia) medium supplemented with 10% fetal bovine serum (FBS, Gibco, Amercia), and NADK- stable knockdown cell lines received additional puromycin to a final concentration of 2.5 µg/mL. HEK293T cells were maintained in DMEM (Gibco, Amercia) medium supplemented with 10% FBS. All cell lines were cultured in a humidified incubator (Thermo Fisher Scientific) at 37 °C with 5% CO_2_. The information of inhibitors or reagents used to treat the cells are as follows: ferroptosis inducer Erastin (Selleck, 0-9 µM), ferroptosis inducer RSL3 (Selleck, 0-0.5 µM), necroptosis inhibitor GSK'872 (Selleck, 10 µM), apoptosis inhibitor Z-VAD-FMK (Selleck, 5 µM), ferroptosis inhibitor Liproxstatin-1 (Selleck, 10 µM), ferroptosis inhibitor Deferoxamine (DFO, Selleck, 20 µM), and NADPH (Beyotime, 200 µM).

### Construction of stable knockdown cell lines

Short hairpin RNA (shRNA) plasmid against NADK, *pLKO.1-puro-shNADK*, was purchased from Shanghai Generay Biotechnology. The sequences of sense and antisense strand for *NADK* shRNA are as follows: sense: 5′- CCGGGCCTACTGCAGCCGTTCAATTCTCGAGAATTGAA CGGCTGCAGTAGGCTTTTTG-3′; antisense: 5′- CGGATGACGTCGGCAAGTTAAGAGCTCC TTAACTTGCCGACGTCATCCGAAAAACTTAA-3′. HEK293T cells were co-transfected with *pLKO.1- puro-shNADK* plasmid and two lentiviral packaging plasmids *psPAX2* and *pMD2.G* using Lipofectamine2000 (Thermo Fisher Scientific, America) according to the manufacturer's protocol. The cell culture supernatant was collected every 24 h for two days, then filtered, mixed with fresh complete medium at a 1:1 ratio and used to culture H1299 and A549 cells with polybrene at a final concentration of 5 µg/mL. After 24 h, the medium was replaced with puromycin (5 µg/mL) -containing complete medium until no floating dead cells were observed, followed by maintenance with 2.5 µg/mL puromycin. Knockdown efficiency was verified by quantitative real-time PCR** (**qPCR) and Western blotting.

### siRNAs/FSP1 and transfection

siRNAs targeting *NADK* were designed and supplied by Shanghai GenePharma. The FSP1 overexpression plasmid(pLV3-CMV-AIFM2(human)-3xFLAG-Puro) was purchased from Wuhan Miaoling Biotechnology Co., Ltd. The sequences of *NADK* siRNAs are as follows: si*NADK*-1, sense: 5′-CCAUCAUAGCCACUCCUU UTT-3′; antisense: 5′-AAAGGAGUGGCUAUGAUGGTT-3′. si*NADK*-2, sense: 5′-GGAAGCGG UGACCCAGGAATT-3′; antisense: 5′-UUCCUGGGUCACCGCUUCCTT-3′. siNC, sense: 5′-UUCUCCGAACGUGUCACGUTT-3′; antisense: 5′-ACGUGACACGUUCGGAGAATT-3′. siRNAs/FSP1 were transfected into H1299 and A549 cells using Lipofectamine2000 (Thermo Fisher Scientific, America) according to the manufacturer's protocol. After transfection for 24–48 h, corresponding experiments were conducted.

### RNA isolation, reverse transcription, and qPCR

Total RNA was extracted using TRIzol Reagent (Invitrogen, Thermo Fisher Scientific, Amercia) and quantified by NANODROP ONE (Thermo Fisher Scientific, Amercia). One µg of total RNA was then reverse transcribed to cDNA using the TAKARA RR047A PrimeScript RT reagent Kit with gDNA Eraser (Perfect Real Time). Gene expression levels were measured using a qPCR machine (BIOER, FQD-96A, HangZhou) with MonAmp™ ChemoHS qPCR Mix (Monad, ShangHai). The relative expression level of *NADK* was calculated using the 2^−ΔΔCt^ method with *GAPDH* as the internal control. The primer sequences are as follows: *NADK* forward: 5′-ACCTGAAGCAAGGAACACAGC-3′; *NADK* reverse: 5′-AGCGGGTAGCATGAGGTAGT- 3′. *GAPDH* forward: 5′-GCTGAGAACGGGAAGCTTGT-3′; *GAPDH* reverse: 5′ -GCCAGGG GTGCTAAGCAGTT-3′.

### Western blotting

Cells were washed with PBS and lysed by radio-immunoprecipitation assay (RIPA) buffer (Beyotime, Shanghai) containing Phenylmethanesulfonyl fluoride (PMSF). Lysates were centrifuged and the supernatant was collected. Protein concentration was determined by bicinchoninic acid (BCA) protein assay kit (Beyotime, Shanghai). Proteins were separated by sodium dodecyl sulfate–polyacrylamide gel electrophoresis (SDS-PAGE) and transferred to a nitrocellulose membrane. The membrane was blocked with 5% bovine serum albumin (BSA) and incubated with primary antibodies overnight at 4 °C. The following day, the membrane was incubated with HRP-conjugated secondary antibodies for 2 h at room temperature, and the signals were detected using an ECL solution by BIO-RAD ChemiDocTM XRS^+^. The information of primary antibodies are as follow: NADK, dilution at 1:1000 (Proteintech, WuHan); FSP1, dilution at 1:1000 (Abclonal, WuHan); GAPDH, dilution at 1:10,000 (Proteintech, WuHan). The secondary antibody was diluted at 1:2000 (Abbkine, WuHan).

### Immunohistochemistry (IHC)

Tissue microarrays were purchased from Shanghai Outdo Biotech Co.Itd. After depara- ffinization and rehydration, antigen retrieval was performed using citrate or Tris–EDTA (TE) buffer at 100 °C for 30 min. Sections were blocked with 3% goat serum and incubated with primary antibodies against NADK (Proteintech, WuHan, 1: 50) or FSP1 (Abclonal, WuHan, 1: 200) overnight at 4 °C. The following day, after washing with TBST, sections were incubated with secondary antibodies at 37 °C for 1 h. DAB was used for color development, and hematoxylin was used for nuclear counterstaining. Expression levels of NADK and FSP1 were assessed by pathologists. Scoring was based on intensity and percentage of positive cells, with scores ≥ 6 classified as high expression.

### Cell viability assay

Cells were seeded at 1500 cells/well in a 96-well plate. At the indicated times, CCK-8 solution (MedChemExpress, America) was added to each well (1:9 ratio with the fresh medium) follow by incubation at 37 °C for one hour. Absorbance at 450 nm was measured using a microplate reader.

### Measurement of malondialdehyde (MDA) level

Cells were lysed and MDA levels were measured using Lipid Peroxidation MDA Assay Kit (Beyotime, Shanghai) following the manufacturer's instructions. Samples were heated in a boiling water bath for 30 min, and the absorbance at 532 nm was measured. Protein concentration was determined by BCA assay for normalization.

### NADP(H) and NADP + /NADPH ration measurement

NADP(H) and NADP^+^/NADPH ration were detected by NADP^+^/NADPH Assay Kit with WST-8 (Beyotime, Shanghai) following the manufacturer's instructions. Briefly, cells were washed by PBS and lysed in NADP^+^/NADPH extraction buffer. After centrifugation, supernatants were used to measure the total NADP(H) and NADPH levels at 450 nm. The Protein concent- ration was used for normalization.

### Detection of ROS with DCFH-DA

The intracellular ROS level was detected with a Reactive Oxygen Species Assay Kit (Beyo time, Shanghai) as per the manufacturer's instructions. Briefly, cells were incubated with DCFH- DA probe diluted in serum-free medium to a final concentration of 10 µM. After incubation at 37 °C for 30 min, fluorescence was detected using an inverted fluorescence microscope.

### Detection of iron (Fe^2+^) with FerroOrange

Cells were washed and incubated with FerroOrange probe (Dojindo, Japan) diluted in serum-free medium to a final concentration of 1 µM. After incubation at 37 °C for 30 min, fluorescence was detected using an inverted fluorescence microscope.

### Propidium iodide (PI) staining

Cells were stained with PI (2.5 μg/ml) at 37 °C for 15 min, followed by DAPI staining (5 µg/mL) at room tempurature for 10 min. After washing, cells were observed under an inverted fluorescence microscope.

### Colony formation assay

Cells were seeded at 800 cells/well in six-well plates and cultured in medium supplemented with 10% serum. The medium was changed every 3–5 days. After 14 days, colonies were fixed with methanol, stained with crystal violet (Beyotime, Shanghai), photographed, and counted.

### Xenograft experiments in nude mice

NADK-knockdown and control A549 cells (1 × 10^6^/mouse) were subcutaneously injected into the left and right armpits of 6-week-old NU/NU nude mice (Charles river, Beijing), respectively. After 50 days, mice were euthanized, and tumors were photographed and weighed. This animal experiment was approved by the Animal Care and Use Committee of Dalian Medical University.

### Statistical analysis

Data were analyzed using SPSS 20.0 or GraphPad Prism 8, and represented as mean ± SD. The statistical difference between two groups was analyzed using two-tailed student’s t-test. Survival analysis was performed using the Kaplan–Meier method with log-rank test for statistical analysis. The association between NADK expression and clinicopathological features was analyzed using the chi-square test. The correlation between NADK and FSP1 IHC scores was evaluated using Spearman's rank correlation coefficient. A *p* value < 0.05 was considered statistically significant (*p < 0.05, ** p < 0.01, *** p < 0.001).

## Results

### NADK is highly expressed in LUAD and associates with poor prognosis

**Fig. 1:**
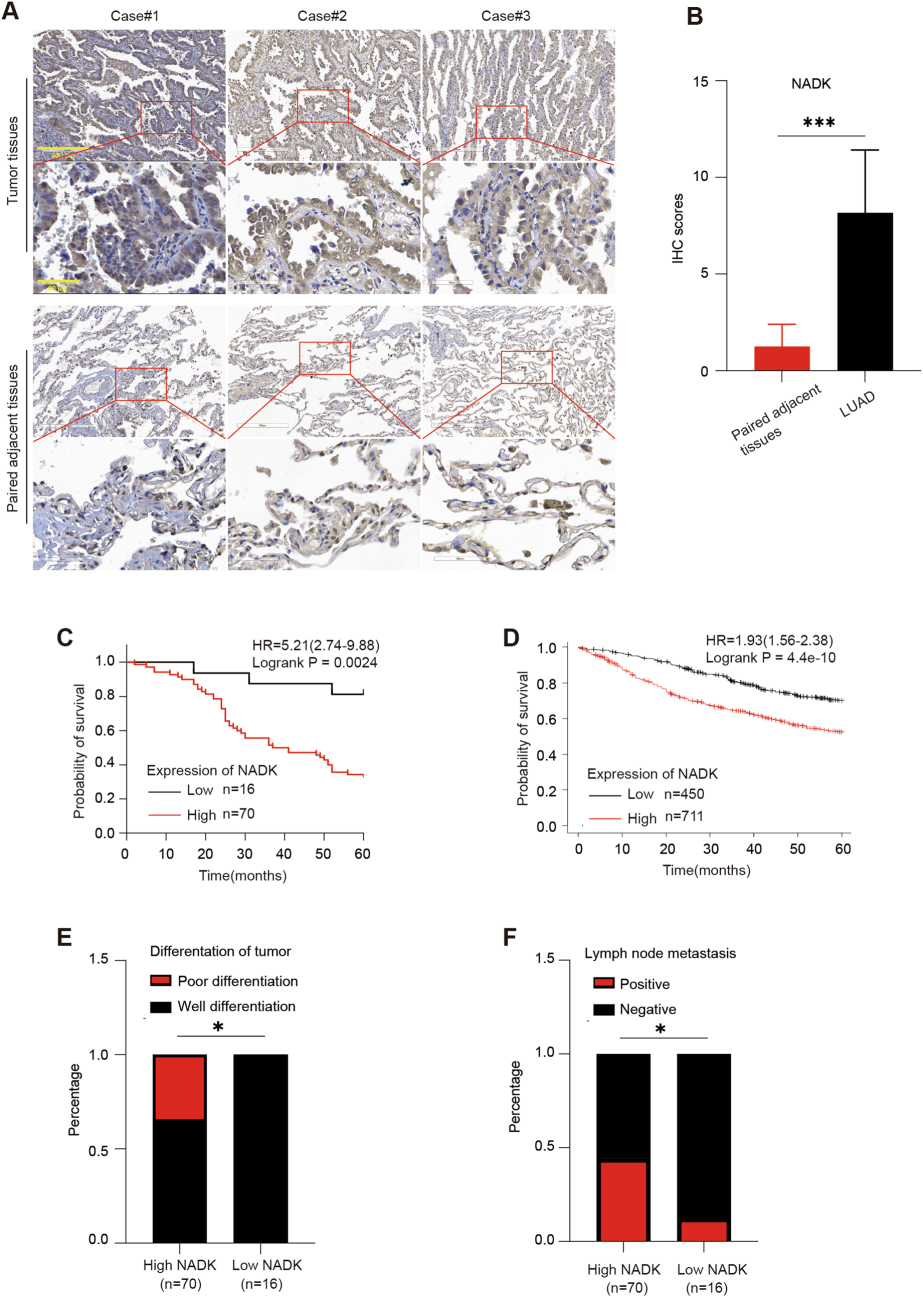
Elevated NADK expression in LUAD correlates with poor prognosis. **A** IHC analysis of NADK expression in tumor tissues and adjacent non-cancerous tissues from 86 LUAD patients. **B** IHC scores of NADK expression in tumor tissues and adjacent non-cancerous tissues of LUAD patients. **C** Kaplan–Meier survival curves comparing the 5-year survival rates of LUAD patients with high (*n* = 70, IHC score ≥ 6) versus low (*n* = 16, IHC score < 6) NADK expression among 86 patients. **D** Survival analysis using the Kaplan–Meier plotter database (www.kmplot.com) to delineate the 5-year survival difference between high and low NADK expression groups in 1161 LUAD patients. **E, F** The correlation between NADK expression with tumor differentiation (**E**) and lymph node metastasis (**F**). Pathological grade ≤ 2 was classified as well differentiation group, while pathological grade > 2 was defined as poor differentiation group

To detect the expression of NADK in LUAD, we performed immunohistochemical staining (IHC) on a LUAD tissue microarray and found that the expression of NADK was significantly evaluated in LUAD tissues as compared to adjacent non-cancerous tissues (Fig. 1A, B). Kaplan–Meier survival curve analysis showed that patients with high levels of NADK expression (*n* = 70, IHC score of 6 or higher) had a worse 5-year survival rate than those with low NADK expression (*n* = 16, IHC score below 6), which was consistent with the results obtained from the Kaplan–Meier Plotter database (www.kmplot.com) (Fig. 1C, D). Moreover, patients with high NADK expression (pathological grade greater than grade II) exhibited a poor degree of tumor differentiation relative to those with low NADK expression (pathological grade less than or equal to grade II), and are more prone to lymph node metastasis (Fig. 1E, F). Collectively, these findings indicate that NADK is highly expressed in LUAD, and elevated NADK expression is associated with poor tumor differentiation, lymph node metastasis as well as poor prognosis in LUAD patients.

### Knockdown of NADK inhibits the proliferation of LUAD cells in vivo and vitro

**Fig. 2:**
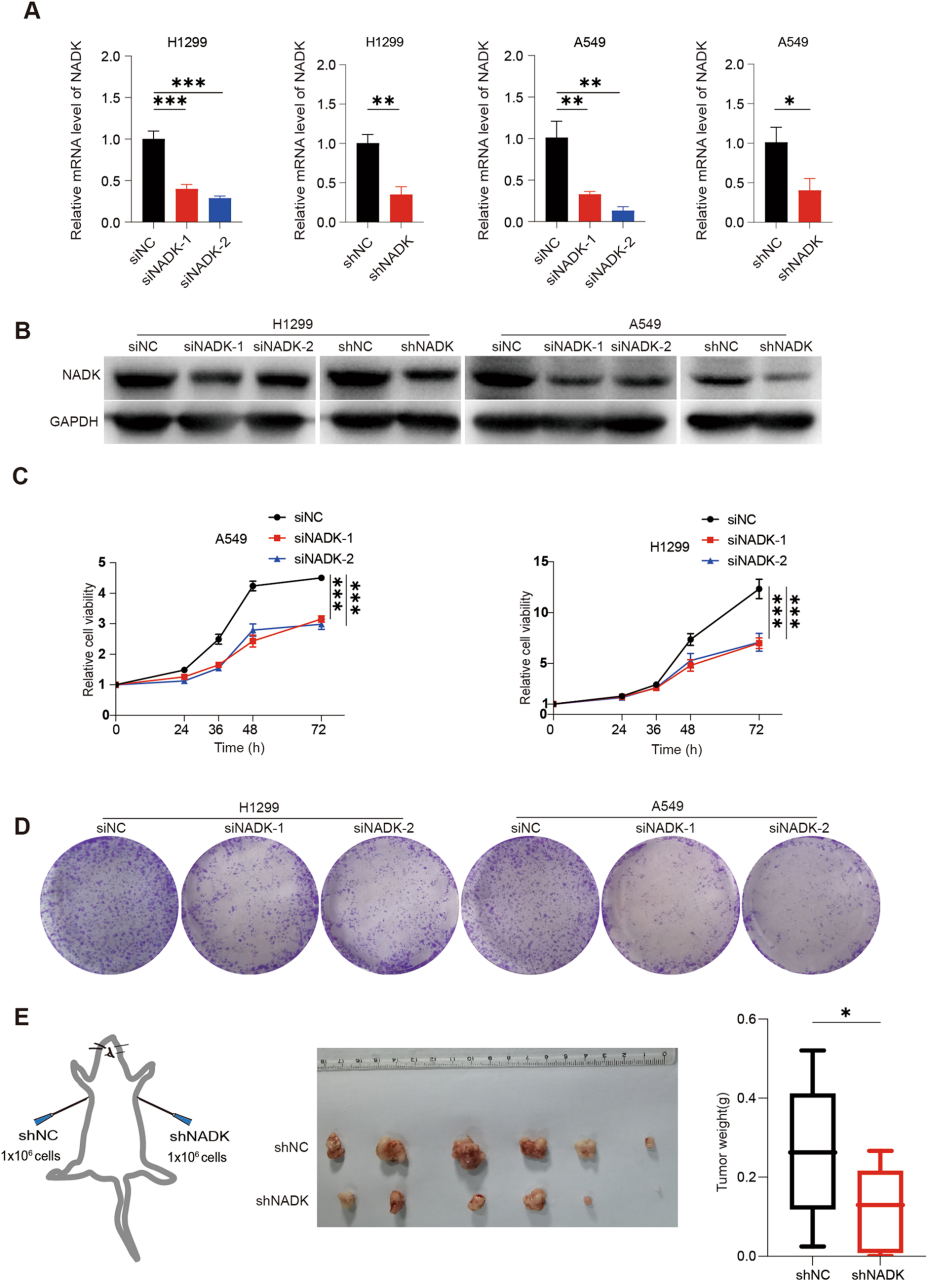
Knockdown of NADK inhibits the growth of LUAD cells in vivo and in vitro. **A** The qPCR validation of NADK mRNA levels in NADK-transient or stable knockdown and control LUAD cells. **B** Western blotting validation of NADK protein levels in NADK-transient or stable knockdown and control LUAD cells. **C** Cell viability of NADK-transient knockdown and control LUAD cells was assessed using CCK-8 assays at the indicated time points, with results normalized to the 0-h point (12 h of seeded cells was recorded as 0-h point). **D** Colony formation assays were performed in NADK-transient knockdown and control LUAD cells. **E** Xenograft experiments in nude mice were performed to investigate the effect of NADK knockdown on the proliferation ability of LUAD cells in vivo

To investigate the role of NADK in LUAD, we knocked down NADK expression in lung adenocarcinoma cell lines A549 and H1299 by transient transfection of siRNA or lentivirus- mediated deliver shRNA techniques. Knockdown efficiency was detected by qPCR and Western blotting, which showed a significantly decrease in NADK mRNA and protein levels (Fig. 2A, B). CCK-8 assays were then performed and demonstrated that the viability of A549 and H1299 cells was significantly inhibited upon NADK knockdown (Fig. 2C). Colony formation assays further showed that knockdown of NADK led to smaller and fewer colonies, indicating an obviously decrease in colony formation ability (Fig. 2D). In agreement with these in vitro results, xenograft experiments in nude mice revealed that tumors formed by NADK-knockdown A549 cells were smaller than those formed by control cells (Fig. 2E). Taken together, these results suggest that NADK is crucial for the proliferation of LUAD cells, and knockdown of NADK can inhibit LUAD cell proliferation both in vivo and vitro.

### Knockdown of NADK promotes the ferroptosis of LUAD cells

**Fig. 3:**
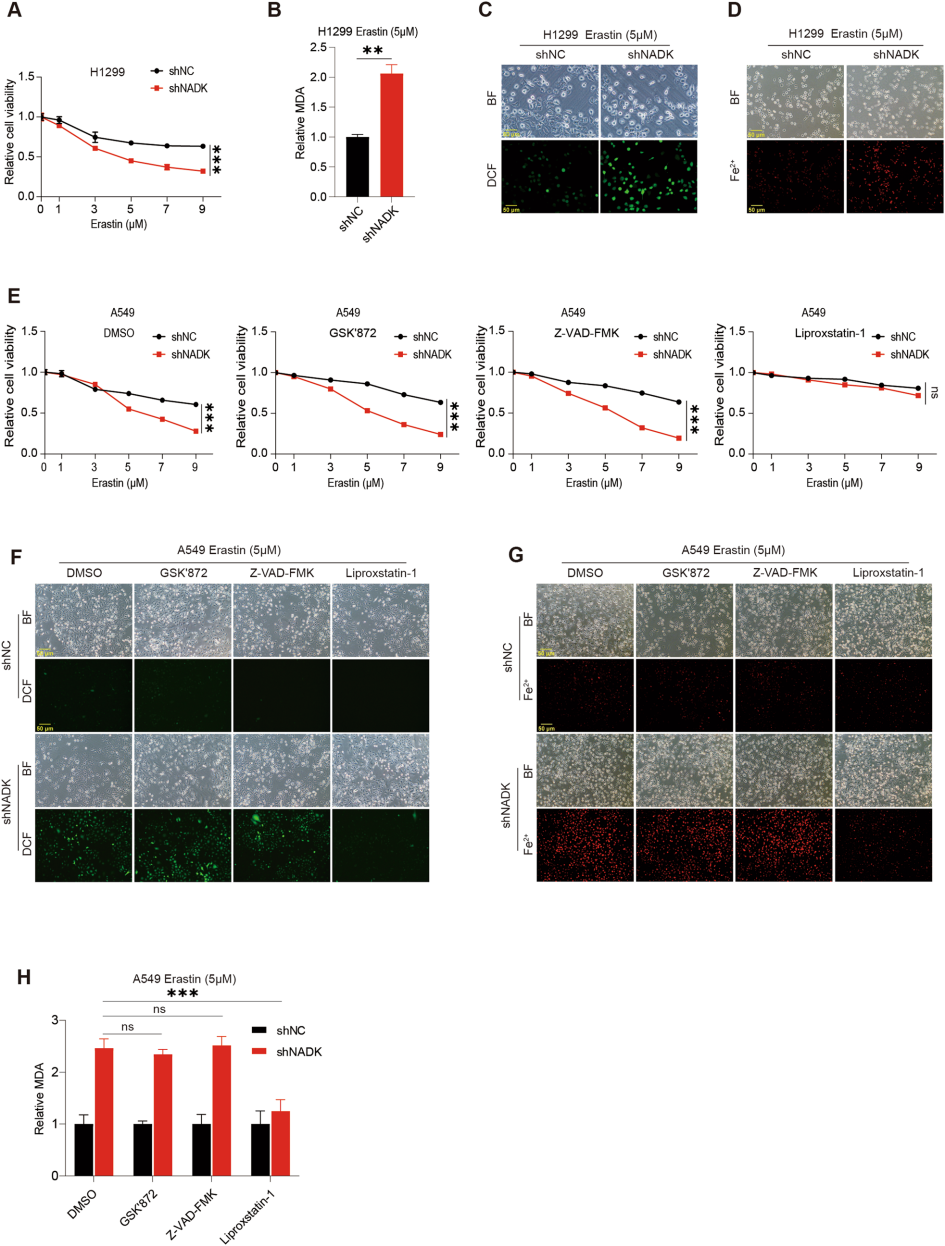
Knockdown of NADK promotes the ferroptosis of LUAD cells. **A** Cell viability of NADK-stable knockdown and control H1299 cells was assessed by CCK-8 assays, after 24 h of treatment with varying concentrations of Erastin. Statistical significance was analyzed at 9 μM concentration. **B** MDA levels of NADK-stable knockdown and control H1299 cells were measured after 24 h of treatment with 5 μM Erastin. **C**, **D** Intracellular ROS (**C**) and Fe^2+^ (**D**) levels of NADK-stable knockdown and control H1299 cells were detected using DCFH-DA probe and FerroOrange probe, respectively, after 24 h of treatment with 5 μM Erastin. **E** Cell viability of NADK-stable knockdown and control A549 cells was assessed with CCK-8 assays, after 24 h of treatment with varying concentrations of Erastin in combination with DMSO, GSK'872 (10 µM), Z-VAD-FMK (5 µM), and Liproxstatin-1 (10 µM), respectively. Statistical significance was analyzed at 9 μM concentration. **F**–**H** Intracellular ROS (**F**), Fe^2+^ (**G**), and MDA (**H**) levels of NADK-stable knockdown and control A549 cells were measured after 24 h of treatment with 5 μM Erastin in combination with DMSO, GSK'872 (10 µM), Z-VAD-FMK (5 µM), and Liproxstatin-1 (10 µM), respectively

To evaluate the effect of NADK on ferroptosis in LUAD cells, we treated NADK- knockdown H1299 cells with a series of different concentrations of ferroptosis inducer (FIN) Erastin or RSL3 for 24 h. CCK-8 assays showed that the viability of NADK-knockdown H1299 cells was significantly reduced upon Erastin or RSL3 treatment compared to control cells (Fig. 3A, S1A and S2A), suggesting that knockdown of NADK promotes the FIN-induced ferroptosis. To confirm this result, we detected MDA levels and found that MDA levels were increased in NADK-knockdown H1299 cells upon Erastin or RSL3 treatment (Fig. 3B, S1B and S2B). Moreover, DCFH-DA probe staining and FerroOrange probe staining revealed that knockdown of NADK led to an increase in ROS (Fig. 3C, S1C and S2C) and Fe^2+^ (Fig. 3D). To further validate the role of NADK in ferroptosis, we treated NADK-knockdown A549 cells with Erastin or RSL3 in combination with DMSO, necroptosis-specific inhibitor GSK'872, apoptosis-specific inhibitor Z-VAD-FMK, and the ferroptosis-specific inhibitor Liproxstatin-1 or Deferoxamine (DFO), respectively. We observed that only Liproxstatin-1 and DFO could specifically restore the decreased cell viability induced by Erastin or RSL3 in NADK-knockdown A549 cells, whereas GSK'872 and Z-VAD-FMK could not (Fig. 3E, S1D and S2D). Similarly, Liproxstatin-1 or DFO specifically reversed the increase in ROS, MDA and Fe2^+^ upon Erastin or RSL3 treatment in NADK-knockdown A549 cells (Fig. 3F–H,S1E, F and S2 E, F). In summary, these results strongly demonstrate that knockdown of NADK increases the sensitivity of LUAD cells to ferroptosis.

### NADK regulate ferroptosis in LUAD cells by maintaining NADPH levels

**Fig. 4:**
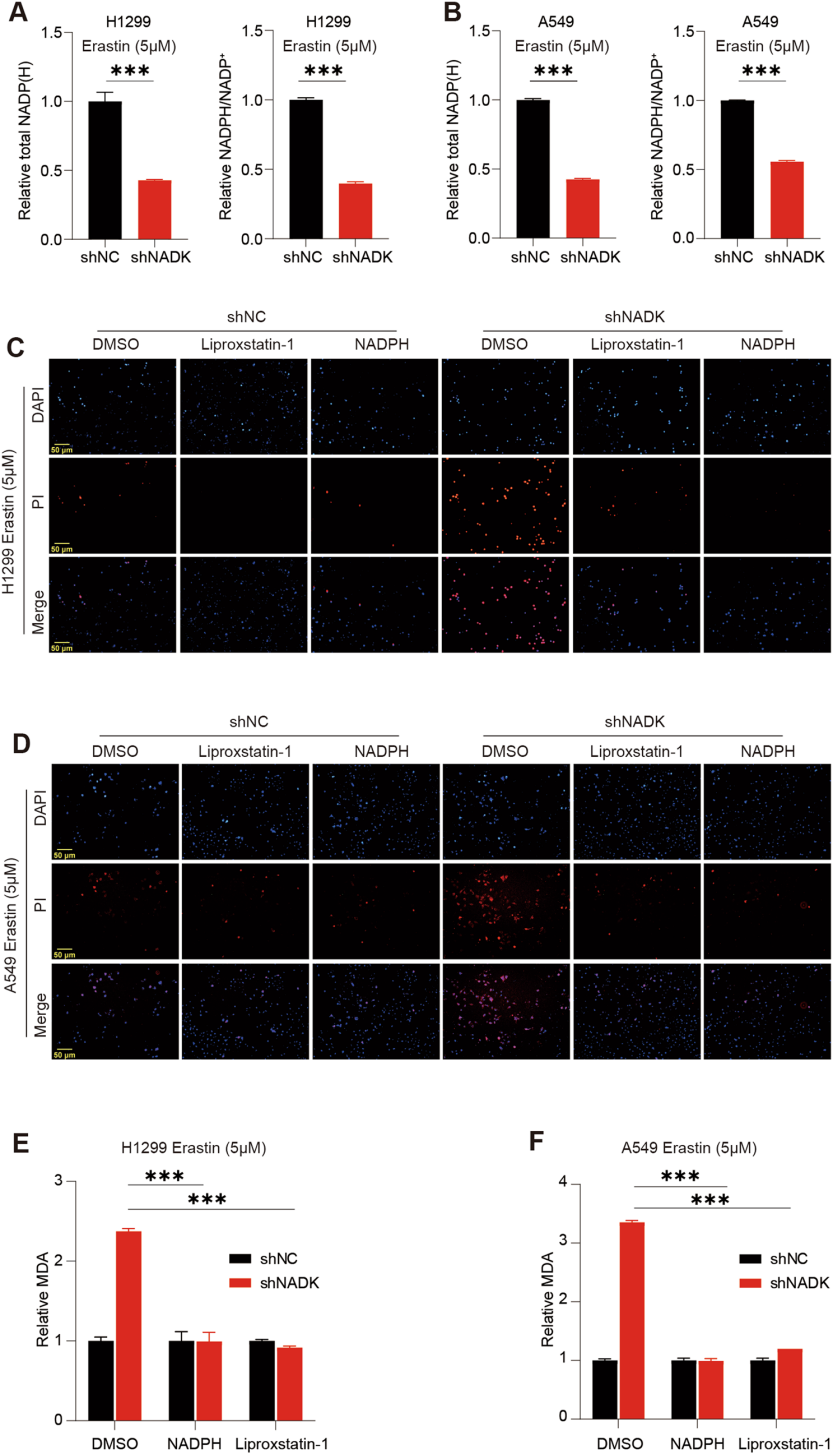
Knockdown of NADK enhances Erastin-induced ferroptosis in LUAD cells by downregulating NADPH levels. **A**, **B** Total levels of NADP^+^ and NADPH as well as the ratio of NADPH/NADP^+^ in NADK-stable knockdown and control H1299/A549 cells were measured using NADP^+^/NADPH Assay Kit with WST-8 after 24-h treatment with 5 μM Erastin. **C**, **D** Cell death of NADK-stable knockdown and control H1299/A549 cells were assessed by PI staining, after 24 h of treatment with 5 μM Erastin in combination with DMSO, Liproxstatin-1, and NADPH, respectively. **E**–**F** Intracellular MDA levels of NADK-stable knockdown and control H1299(**E**)/A549(**F**) cells were measured, after 24 h of treatment with 5 μM Erastin in combination with DMSO, NADPH, and Liproxstatin-1, respectively

As NADK can catalyze NAD^+^ to generate NADP^+^, NADK plays an important role in maintaining the levels of NADP^+^ and NADPH. Indeed, after NADK was knocked down in LUAD cells, the overall levels of intracellular NADP^+^ and NADPH were significantly decreased, and the NADPH/NADP^+^ ratio was also declined significantly (Fig. 4A, B), confirming that NADK maintains the levels of NADP^+^ and NADPH in cells. Thus, we speculated that NADK might regulate ferroptosis by controlling the production of NADP^+^ and NADPH. To confirm this hypothesis, we added NADPH to NADK-knockdown H1299/A549 cells upon treatment of Erastin for 24 h, with Liproxstatin-1 as a positive control. PI staining showed that knockdown of NADK led to an obviously increase in PI-positive cells, while both Liproxstatin-1 and NADPH could reverse this effect (Fig. 4C, D). Consistently, like Liproxstatin-1, NADPH could restore the increase in MDA (Fig. 4E, F), ROS (Fig S4 A, B) and Fe^2+^ (Fig S4 C, D), upon Erastin treatment in NADK-knockdown H1299/A549 cells. Overall, these findings indicate that knockdown of NADK increases the sensitivity of LUAD cells to ferroptosis by reducing NADPH levels.

### NADK regulates FSP1 levels in LUAD cells

**Fig. 5:**
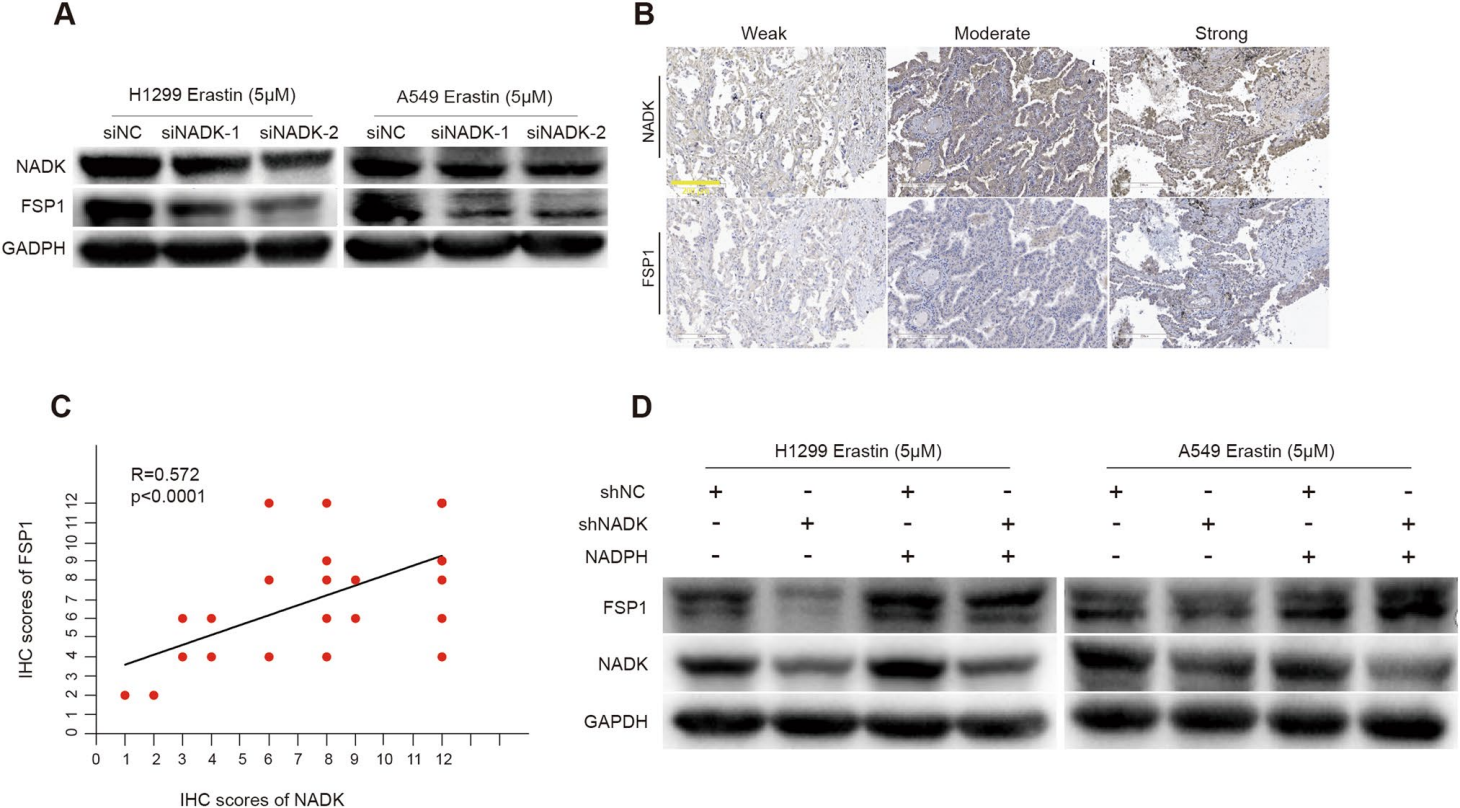
NADK regulates FSP1 levels in LUAD cells. **A** Western blotting analysis of NADK and FSP1 protein levels in NADK-transient knockdown and control H1299/A549 cells following 24 h of treatment with 5 μM Erastin. **B**, **C** IHC analysis of NADK and FSP1 expression in LUAD tissue arrays, with Spearman analysis of the correlation between NADK and FSP1 IHC scores. **D** Western blotting analysis of NADK and FSP1 protein levels in NADK-stable knockdown and control H1299/A549 cells, after 24 h of treatment with 5 μM Erastin together with NADPH

To explore the ferroptosis-related genes regulated by NADK, we employed the GEPIA(http://gepia.cancer-pku.cn/) and Timer2.0 (http://timer.comp-genomics.org/timer/) databases to investigate the correlation between NADK and five key ferroptosis-related genes: FSP1(also called AIFM2), GPX4, DHODH, GCH1, and SLC7A11(Li et al. 2016, 2017, 2020; Tang et al. 2017). Pearson correlation analysis revealed that the association between NADK and FSP1 was the most significant across both databases. (Timer2.0: R = 0.259, p = 2.54e^−09^; GEPIA: R = 0.29, p = 4.3e^−11^)(Fig S3).Furthermore, we found that knockdown of NADK in LUAD cells resulted in a decrease in FSP1 expression upon Erastin treatment, as determined by Western blotting assays (Fig. 5A). We further examined the expression of FSP1 in LUAD tissue microarray and analyzed the correlation between NADK and FSP1. The results showed that there was a significantly positive correlation between the expression of FSP1 and NADK in LUAD tissues (Fig. 5B, C). Interestingly, addition of NADPH to NADK-knockdown H1299/A549 cells effectively restored the reduction in FSP1 expression upon Erastin treatment (Fig. 5D). To confirm the role of NADK/FSP1 in ferroptosis, we transiently transfected FSP1-containing vectors into NADK-knockdown H1299/A549 cells. The overexpression efficiency of FSP1 was confirmed by Western blot assay (Supplementary Figure S5A). PI staining revealed that overexpression of FSP1 led to an obviously decrease in PI-positive cells in NADK-knockdown LUAD cells (Supplementary Figure S5B). Also, overexpression of FSP1 reversed the increased levels of ROS and MDA caused by knockdown of NADK (Supplementary Figure S5C, D and E). These findings suggest that NADK may regulate ferroptosis via the NADPH/FSP1 axis.

## Discussion

A prominent feature of lung cancer is that it originates from high-oxygen content regions and thus exhibits an enhanced antioxidative capabilities. The imbalance in redox homeostasis, which leads to ROS mediated cellular membrane damage, is a critical stage in the ferroptosis (Jiang et al. 2021; Yao et al. 2021). It has been reported that NFS1 is upregulated in lung cancer, thereby conferring cancer cells resistance to high oxygen tension by inhibiting the ferroptosis (Alvarez et al. 2017). Thus, resistance to ferroptosis might be one of the contributing factors to the high mortality rate of lung cancer.

The mechanisms of tumor resistance to ferroptosis are intricate. For example, in human fibrosarcoma cells, GPX4 utilizes GSH to eliminate lipid peroxides and plays an important role in counteracting ferroptosis, while depletion of GPX4 or GSH enhances the sensitivity of fibrosarcoma cells to Erastin-induced ferroptosis (Yang et al. 2014). In colorectal cancer, lncRNA LINC01606 enhanced the expression of SCD1 and activated the Wnt/β-catenin pathway, which formed a positive feedback loop with LINC01606 to inhibit ferroptosis (Luo et al. 2022)**.** Lung cancer also exhibits multiple regulatory mechanisms of ferroptosis. TFEB has been reported to promote lysosome-mediated GPX4 degradation, thus enhancing β-Elemene- induced ferroptosis in NSCLC cells (Zhao et al. 2023). FSP1, also known as AIFM2, was initially identified to induce caspases and P53-independent apoptosis in HEK293T cells (Wu et al. 2002). Recently, FSP1 has been shown to possess anti-ferroptosis function in various cancers (Gong et al. 2023; Müller et al. 2023; Yang et al. 2022; Zheng et al. 2022), including NSCLC (Kim et al. 2023b; Yuan et al. 2023). FSP1 can utilize NAD(P)H and oxidized CoQ_10_ as substrates to generate NAD(P)^+^ and reduced CoQ_10_, thus inhibiting ferroptosis in GPX4-depleted NSCLC cells (Bersuker et al. 2019). FSP1 inhibitors have a strong synergistic effect with GPX4 inhibitors (Doll et al. 2019). Hence, FSP1/CoQ_10_/NAD(P)H is an independent but functionally synergistic ferroptosis protection system from GPX4/GSH/GSSG.

NADK is a key enzyme that catalyzes the synthesis of NADP^+^ from NAD^+^ and is one of the important sources of NADPH (Hoxhaj et al. 2019; Love et al. 2015; Pollak et al. 2007). It has been reported that NADK-mediated NADPH increase is an important contributor to breast cancer metastasis (Ilter et al. 2023). Moreover, increased NADK activity is also involved in pancreatic ductal adenocarcinoma (PDAC) (Schild et al. 2021). Although less reported in LUAD, NADK has been found to be overexpressed in NSCLC and to enhance NSCLC cell invasion, migration, and lymph node metastasis by activating BMPs/ID1 (Zeng et al. 2023). However, its effect on ferroptosis in lung cancer has not been well-documented. Here, our data further validated that NADK was overexpressed in LUAD tissues relative to adjacent non-cancerous tissues, and high NADK expression was associated with poor tumor differentiation, lymph node metastasis, and poor prognosis in LUAD patients. Knockdown of NADK inhibited LUAD cell proliferation both in vivo and vitro, indicating that NADK promotes the development and progression of LUAD. Furthermore, we demonstrated that knockdown of NADK enhanced the sensitivity of LUAD cells to Erastin or RSL3-induced ferroptosis, which could be restored by a specifically ferroptosis inhibitor.

Moreover, we found that knockdown of NADK led to a decrease in intracellular NADPH abundance. Exogenous supplementation of NADPH could inhibit the Erastin-induced ferroptosis of NADK knockdown LUAD cells, indicating that the catalytic activity of NADK is necessary for its resistance to ferroptosis. Additionally, IHC staining with clinical LUAD samples showed a positive correlation between NADK and FSP1 expression. Knockdown of NADK also led to a decrease in FSP1 expression, which could be restored by exogenous supplementation of NADPH. Collectively, these findings elucidate that NADK can regulate ferroptosis by controlling NADPH and FSP1 levels, providing a novel target and strategy for sensitizing LUAD to ferroptosis.

## Conclusion

Our study reveals that knockdown of NADK promotes the Erastin or RSL3-induced ferroptosis of LUAD cells by down-regulating the NADPH/FSP1 axis, indicating that inhibition of NADK enzymatic activity can synergize with ferroptosis inducers. These findings provide a new insight into the regulatory mechanism of FSP1 in LUAD and a novel theoretical basis for increasing the sensitivity of LUAD to ferroptosis.

### Supplementary Information

Below is the link to the electronic supplementary material.

Supplementary file1 (TIF 10165 KB)

Supplementary file2 (TIF 7173 KB)

Supplementary file3 (TIF 4459 KB)

Supplementary file4 (TIF 13674 KB)

Supplementary file5 (TIF 9682 KB)

## Data Availability

All the data used in the present study are available from the corresponding author upon reasonable request.
