# Supplementary figures and images for: Knockdown of NADK promotes LUAD ferroptosis via NADPH/FSP1 axis
Source: J Cancer Res Clin Oncol. 2024 May 3;150(5):228. doi: 10.1007/s00432-024-05752-z (PMC11068837; doi:10.1007/s00432-024-05752-z)

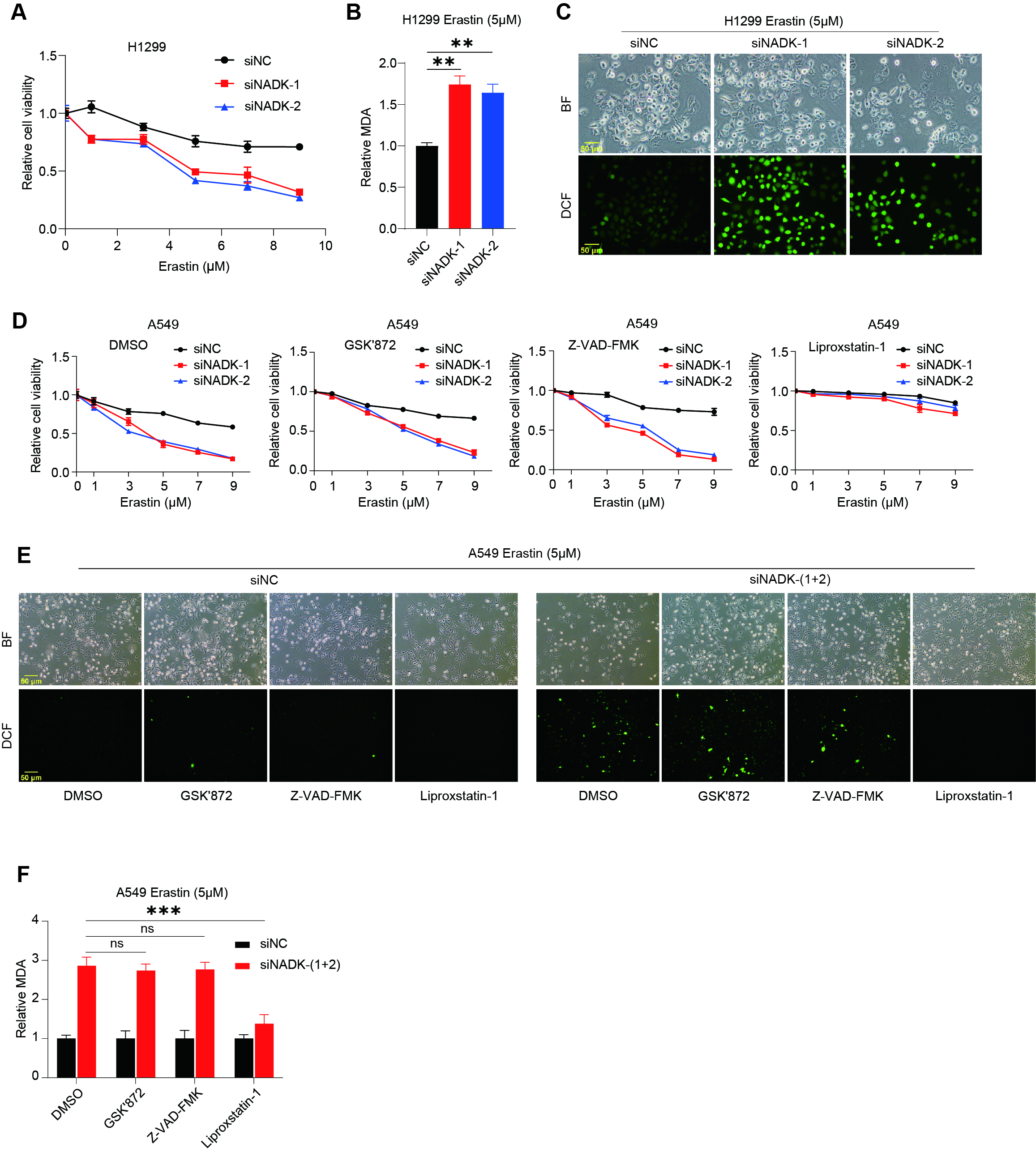

Supplement: Supplementary file 1 — Supplementary file1 (TIF 10165 KB) [file 432_2024_5752_MOESM1_ESM.tif]

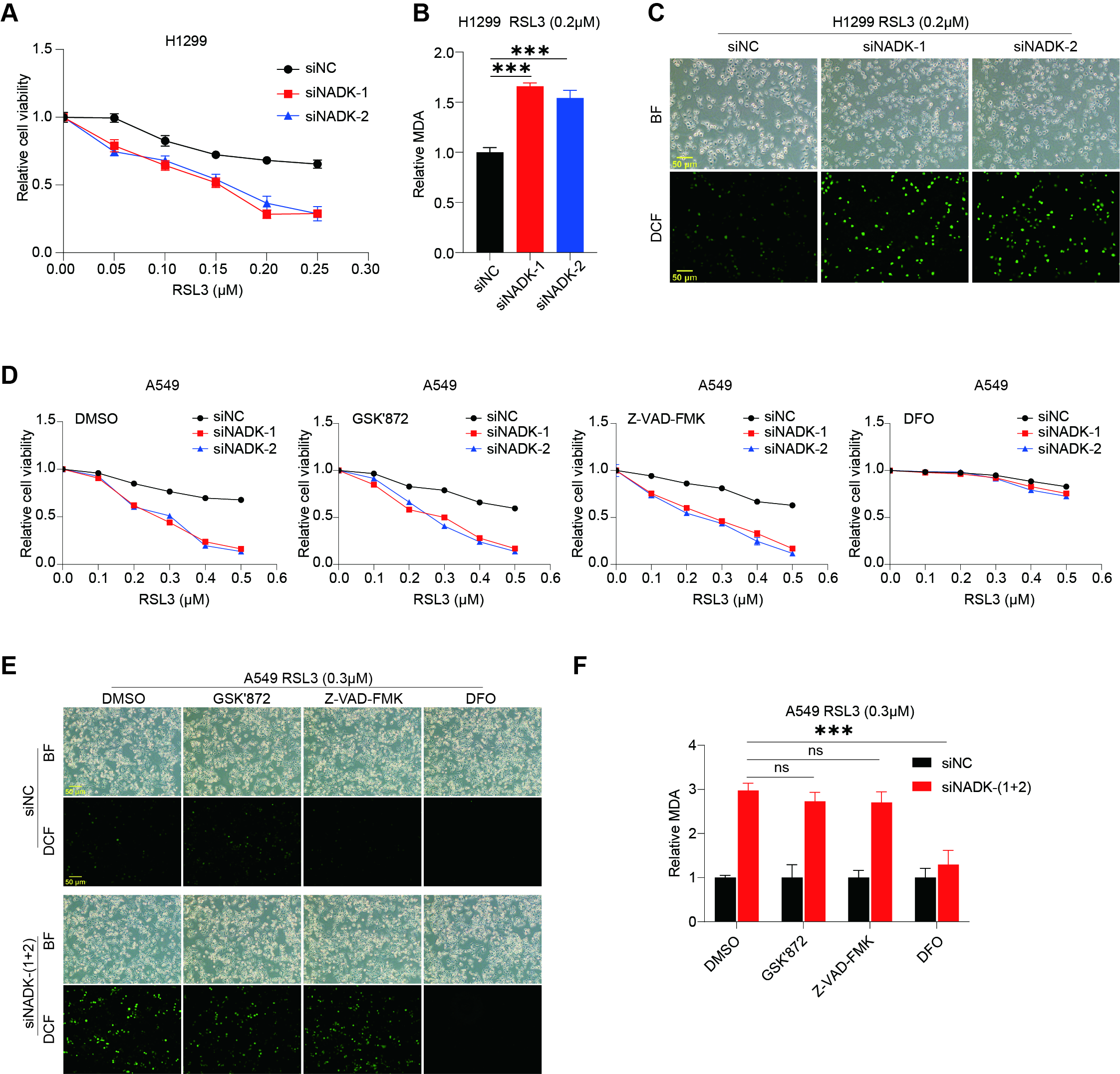

Supplement: Supplementary file 2 — Supplementary file2 (TIF 7173 KB) [file 432_2024_5752_MOESM2_ESM.tif]

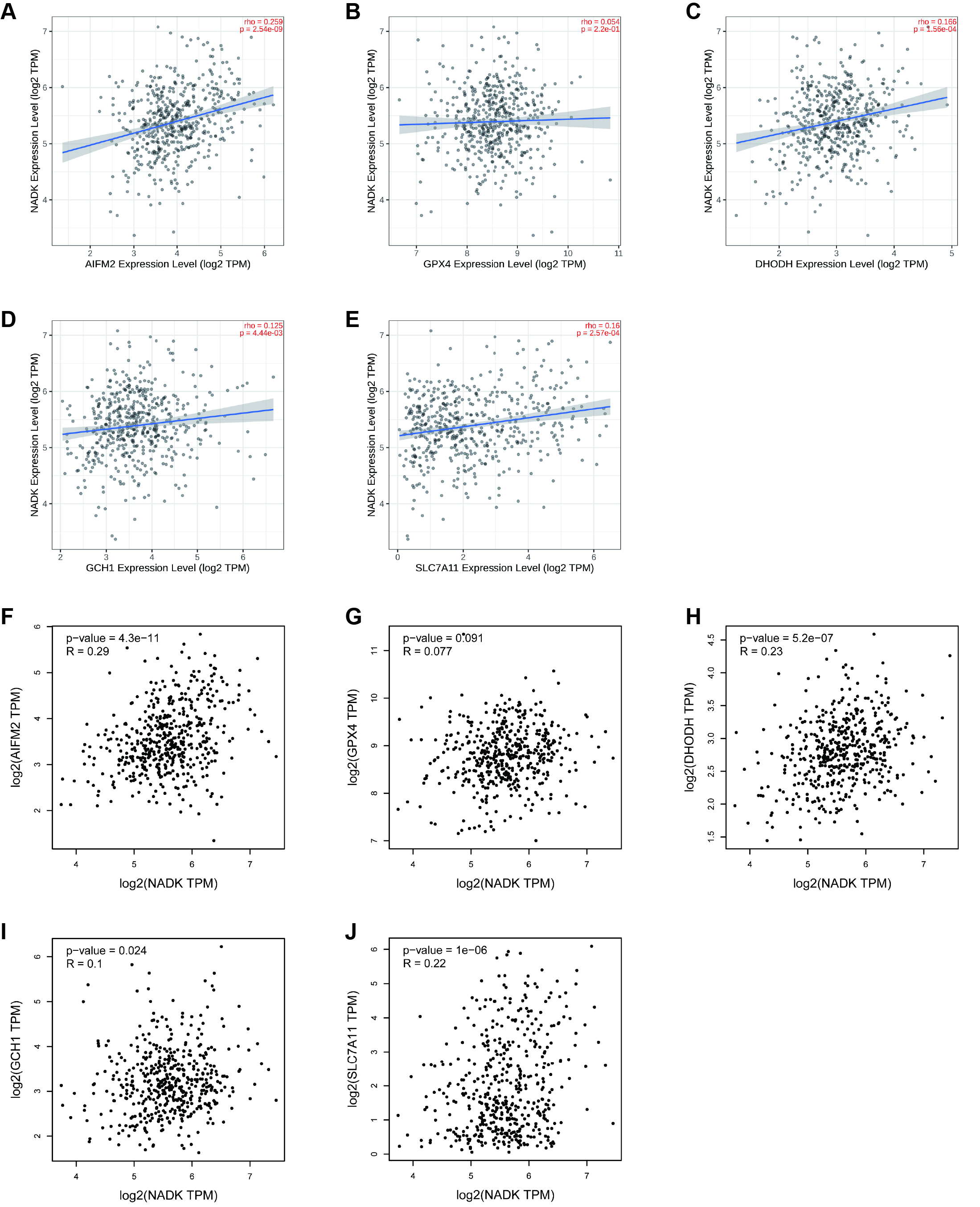

Supplement: Supplementary file 3 — Supplementary file3 (TIF 4459 KB) [file 432_2024_5752_MOESM3_ESM.tif]

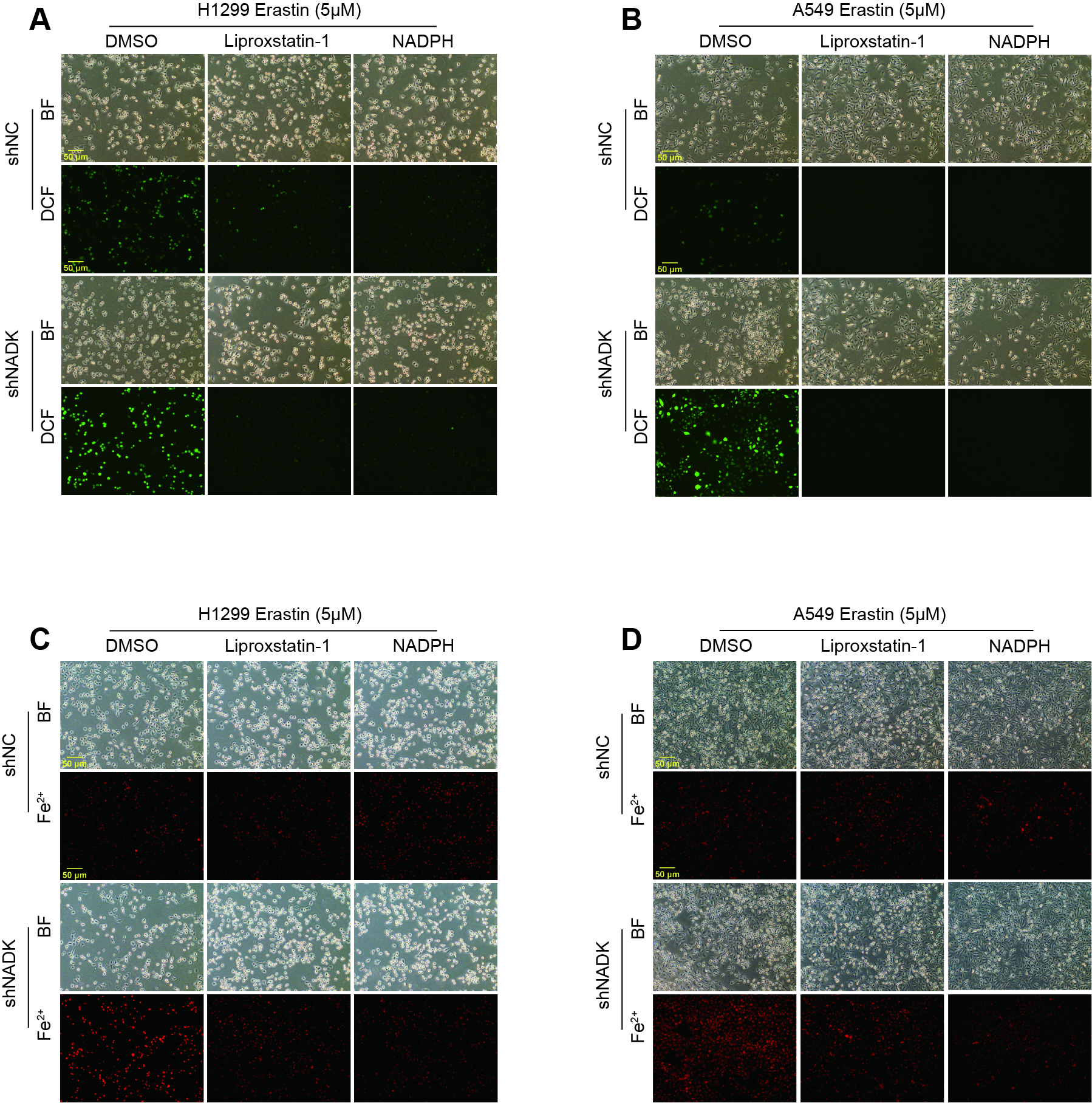

Supplement: Supplementary file 4 — Supplementary file4 (TIF 13674 KB) [file 432_2024_5752_MOESM4_ESM.tif]

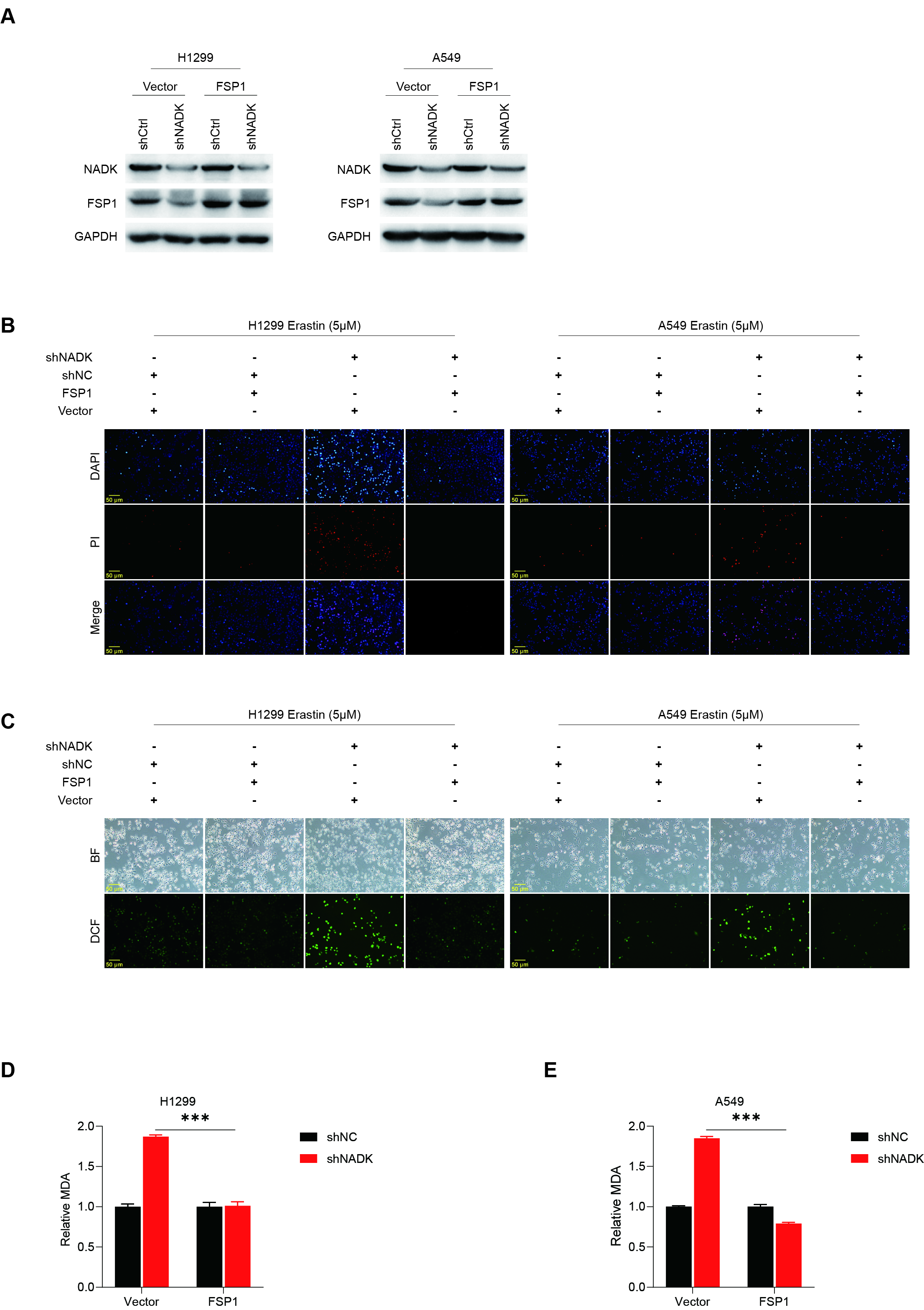

Supplement: Supplementary file 5 — Supplementary file5 (TIF 9682 KB) [file 432_2024_5752_MOESM5_ESM.tif]
